# Deep Sequencing of the Oral Microbiome Reveals Signatures of Periodontal Disease

**DOI:** 10.1371/journal.pone.0037919

**Published:** 2012-06-04

**Authors:** Bo Liu, Lina L. Faller, Niels Klitgord, Varun Mazumdar, Mohammad Ghodsi, Daniel D. Sommer, Theodore R. Gibbons, Todd J. Treangen, Yi-Chien Chang, Shan Li, O. Colin Stine, Hatice Hasturk, Simon Kasif, Daniel Segrè, Mihai Pop, Salomon Amar

**Affiliations:** 1 Center for Bioinformatics and Computational Biology, University of Maryland, College Park, Maryland, United States of America; 2 Department of Computer Science, University of Maryland, College Park, Maryland, United States of America; 3 Bioinformatics Program, Boston University, Boston, Massachusetts, United States of America; 4 Biological Sciences Graduate Program, University of Maryland, College Park, Maryland, United States of America; 5 Department of Epidemiology and Public Health, University of Maryland School of Medicine, Baltimore, Maryland, United States of America; 6 Department of Biology, Boston University, Boston, Massachusetts, United States of America; 7 Department of Biomedical Engineering, Boston University, Boston, Massachusetts, United States of America; 8 The Forysth Institute, Department of Periodontology, Cambridge, Massachusetts, United States of America; 9 Children’s Informatics Program, Harvard-Massachusetts Institute of Technology Division of Health Sciences and Technology, Boston, Massachusetts, United States of America; 10 The McKusick-Nathans Institute for Genetic Medicine, The Johns Hopkins University School of Medicine, Baltimore, Maryland, United States of America; 11 Center for Anti-Inflammatory Therapeutics; Boston University Goldman School of Dental Medicine, Boston, Massachusetts, United States of America; Baylor College of Medicine, United States of America

## Abstract

The oral microbiome, the complex ecosystem of microbes inhabiting the human mouth, harbors several thousands of bacterial types. The proliferation of pathogenic bacteria within the mouth gives rise to periodontitis, an inflammatory disease known to also constitute a risk factor for cardiovascular disease. While much is known about individual species associated with pathogenesis, the system-level mechanisms underlying the transition from health to disease are still poorly understood. Through the sequencing of the 16S rRNA gene and of whole community DNA we provide a glimpse at the global genetic, metabolic, and ecological changes associated with periodontitis in 15 subgingival plaque samples, four from each of two periodontitis patients, and the remaining samples from three healthy individuals. We also demonstrate the power of whole-metagenome sequencing approaches in characterizing the genomes of key players in the oral microbiome, including an unculturable TM7 organism. We reveal the disease microbiome to be enriched in virulence factors, and adapted to a parasitic lifestyle that takes advantage of the disrupted host homeostasis. Furthermore, diseased samples share a common structure that was not found in completely healthy samples, suggesting that the disease state may occupy a narrow region within the space of possible configurations of the oral microbiome. Our pilot study demonstrates the power of high-throughput sequencing as a tool for understanding the role of the oral microbiome in periodontal disease. Despite a modest level of sequencing (∼2 lanes Illumina 76 bp PE) and high human DNA contamination (up to ∼90%) we were able to partially reconstruct several oral microbes and to preliminarily characterize some systems-level differences between the healthy and diseased oral microbiomes.

## Introduction

Understanding the role of microbial communities in human health is emerging as one of the most important and fascinating biomedical challenges of our times [1], [2], [3], [4]. Our body harbors an enormous amount of microbial cells, estimated to exceed the number of human cells by an order of magnitude [5]. These microbes are organized into complex communities specifically adapted to inhabit different niches of the human body, such as the skin, and the respiratory, gastrointestinal, and urogenital tracts. Such ecosystems carry a broad range of functions indispensable for the wellbeing of the host [6]. At the same time, the rise of pathogens within such communities, causing infection and inflammation, constitutes an ongoing challenge in biomedical research. This is especially true in light of the slow rate at which new antibiotics are discovered [7], and the increase in the number of microbes that can resist treatment [8], [9]. In contrast to the traditional view of individual pathogens being responsible for disease onset, recent microbial ecosystem diversity analyses seem to point to a new perspective in which the transition from health to disease is attributed to a shift in the global balance of the microbial flora rather than to the specific appearance of individual pathogens [10], [11], [12], [13]. However, the mechanisms that underlie the connection between disease or infection and the dynamics of the host-associated ecosystems are still poorly understood.

In this work, we focus on the role of the oral microbial ecosystem in periodontal disease. Periodontal disease is the most common infectious disease affecting tooth-supporting structures. Left untreated, periodontitis can lead to, or aggravate existing systemic conditions such as cardiovascular disease, diabetes, pulmonary diseases, and obesity [14], [15], [16]. In dentistry, understanding the changes in the oral microbiome that foretell the early stages of periodontitis and dental caries, the most prevalent chronic oral diseases, may allow the better diagnosis and treatment before the appearance of the telltale clinical manifestations of these diseases (such as tissue damage in periodontal pockets or dental hard tissue loss). The emergence and evolution of antibiotic resistance in periodontal pathogens has affected the therapeutic success rates for this disease [17], [18]. New approaches are urgently needed to help regain control over periodontal disease, and microbiome studies offer a promising new angle of attack. Unraveling the complex interactions that define the oral microbiome is a fundamental, but complex component of this endeavor.

Recent developments in systems biology make it possible to perform quantitative modeling of genome-scale metabolic networks for individual microbial species [19], [20] and have been recently extended to explore small microbial consortia [21], [22], possibly paving the way for future quantitative studies of the microbiome. However, at the ecosystem level, current modeling efforts and quantitative analyses are heavily limited by the unavailability of relevant data. Towards this goal, increasingly accessible metagenomic sequencing approaches hold the promise to enable a global systemic view of the human oral microbiome [1], [4], [23]. Recent advances in sequencing technology are enabling scientists to generate billions of nucleotide bases at a fraction of the cost per base of traditional methods [24]. This deep sequencing has revealed an unexpectedly high diversity of the human oral microbiome: dental plaque pooled from 98 healthy adults comprised about 10,000 microbial phylotypes [25] - an order of magnitude higher than the previously reported 700 oral microbial phylotypes as identified by cultivation or traditional cloning and sequencing [26], [27]. The total diversity of the global oral microbiome can be estimated to be around 25,000 phylotypes [25]. To date, however, we do not know how many of these microbes contribute to periodontal disease, what metabolic functions are key players in the transition from health to disease, or how common or exclusive are the oral microbiomes of unrelated healthy individuals.

Here we combine the collection of whole-community sequencing data with a number of computational analyses to provide a snapshot of the microbial component of periodontal disease at a high resolution. Specifically, we collected subgingival plaque samples from healthy and periodontally affected patients and subjected them to 16S rDNA analysis and deep sequencing in order to explore their microbiome. Our analyses reveal a number of trends in genomic diversity and biological function enrichment during disease that allow us to formulate a novel hypothesis on the nature of periodontal disease. We also demonstrate the power of high-throughput sequencing approaches by reconstructing an unculturable member of the TM7 group, complementing an initial analysis that relied on single cell genomic approaches. We also characterize several regions of variation within one of the dominant members of the oral cavity, *Actinomyces naeslundii*. This paper describes a genomic and metabolic examination of the differences between the healthy and diseased periodontal microbiome.

## Results and Discussion

### A Deep Look at the Oral Microbiome in Health and Disease

Current knowledge of the composition and functional spectrum of the human oral microbiome is limited by the difficulty to culture the majority of microbes that populate the oral cavity. We used deep sequencing technology to overcome this limitation, and produce a substantial genomic data set for the human microbiome under health and periodontal disease conditions. Specifically, we generated 16S rDNA data from five subjects (3 periodontally healthy [H] and 2 chronic periodontitis [P] patients, Table 1). In addition, a total of 495,195 16S rDNA sequences were generated with the 454 FLX sequencing technology, yielding an average of ∼30,000 sequences per sample after removing low-quality sequences (roughly 3-times more sequences per sample than generated in a recent survey of oral microbes [28]). A total of 272,709,876 sequence reads were generated using the Illumina GAII platform, 76 bp, paired-end run (mean library size 207 bp) from the whole metagenome of four of the above-mentioned subjects (H1, H2, P1, and P2; Table 1). The low quality nucleotides were trimmed from all sequences and fragments matching to the human genome reference (NCBI release GRCh37.p1) were removed from further analysis. The level of human DNA contamination varied between different samples averaging ∼87% of the sample, i.e. the oral microbiome represents just one eighth of the entire dataset or a total of 33,681,771 (12.4%) sequences (Table 1). This level of contamination is consistent with that observed in other studies, such as the Human Microbiome Project (manuscript in preparation). Despite the moderate yield (in terms of fraction of microbial sequences in the data-set) our results show that valuable biological insights can be derived from the data, thus indicating that informative and clinically relevant whole-metagenomic analyses of the oral microbiota can be conducted in a cost-effective manner.

**Table 1:** Summary of sample information including high-quality read counts, taxonomic assignment of most abundant genus in each sample, and level of human contamination.

Phenotype	Subject (Tooth)	Clinical	16S rDNA		Dominant genus	Shotgun		Human DNA	Dominant genus
			# Reads	Sample		# Reads	Sample		
Periodontal disease	1(14)	advanced	51,056	P11	*Prevotella*	9.7M	P1	68.86%	*Prevotella*
	1(19)	moderate	20,149	P12	*Fusobacterium*				
	1(30)	moderate	41,355	P13	*Prevotella*				
	2(30)	moderate	46,444	P21	*Prevotella*	4.9M	P2	81.98%	*Prevotella*
Healthy	3(1)	healthy	23,702	H11	*Streptococcus*	12.4M	H1	60.61%	*Streptococcus*
	3(2)	healthy	44,869	H12	*Peptostreptococcus*				
	3(3)	healthy	32,405	H13	*Streptococcus*				
	3(4)	healthy	56,116	H14	*Leptotrichia*				
	4(3)	healthy	6,205	H21	*Streptococcus*	6.7M	H2	89.78%	*Actinomyces*
	4(14)	healthy	35,356	H22	*Actinomyces*				
	4(19)	healthy	14,110	H23	*Neisseria*				
	4(30)	healthy	25,662	H24	*Actinomyces*				
	5(3)	early	12,295	H31	*Fusobacterium*	NA	NA	NA	NA
	5(19)	healthy	30,891	H32	*Kingella*				
	5(30)	healthy	12,605	H33	*Actinomyces*				

The clinical labels represent: ‘healthy’ – healthy periodontal pocket; ‘early’ – early periodontal disease (bleeding under probing but no attachment loss), ‘moderate’ – moderate periodontal disease; ‘advanced’ – advanced periodontal disease. For a description of the clinical parameters used to make these determinations see Materials and Methods. The absence of metagenomic data from subject 5 is indicated with ‘NA’ in the appropriate cells.

### Beyond the Taxonomical View of Periodontitis

**Figure 1:**
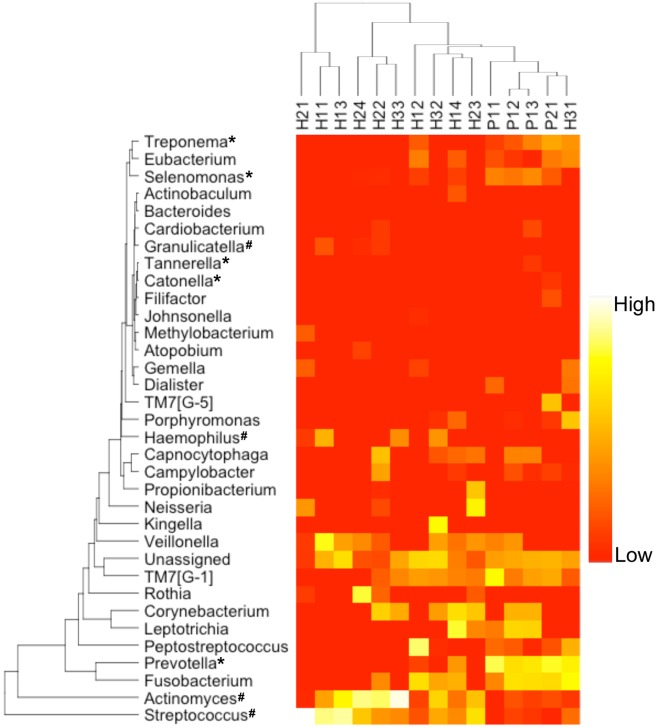
Relative abundance of genera in the samples estimated from 16S rDNA sequencing. * - genus significantly enriched in cases; ∧- genus significantly enriched in controls (p< = 0.05, Metastats [ 30]). Only genera with >1% abundance in at least one sample were included. Colors reflect relative abundance from low (red) to high (white). Sample H31 (control) clusters together with the diseased samples, consistent with clinical observations of early symptoms of periodontal disease.

The standard view of periodontitis, largely based on traditional microbiological approaches, associates the disease with the rise and damaging action of a small set of well-characterized pathogens. A first question we wanted to address using our data is whether, and to what extent, this traditional view still holds from the vantage point of metagenomic sequencing. Taxonomic profiling of the samples, whether derived from targeted 16S rRNA sequencing or from whole-metagenomic data (WGS) (Methods and Figure 1) reveals a community dominated, on average, by the bacterial phyla Firmicutes, Actinobacteria, Bacteroidetes, Fusobacteria and Proteobacteria, consistent with previous studies [28], [29]. Together, these groups account for 80–95% of the entire oral microbiome. At the genus level we identify a total of 55 distinct genera in the 16S rDNA data and 58 distinct genera in the WGS data that are present at an abundance of 0.1% or higher (an additional 73 and 62 rare genera can be found in the 16S rDNA and WGS data, respectively). The most abundant genera comprise previously characterized oral bacteria: *Actinomyces*, *Prevotella*, *Streptococcus*, *Fusobacterium*, *Leptotrichia*, *Corynebacterium*, *Veillonella, Rothia*, *Capnocytophaga, Selenomonas, Treponema*, and TM7 genera 1 and 5.

The TM7 division was prevalent in our samples (11 out of 15 samples contain this division at >2% abundance), averaging 5.7% (standard deviation 7.2) of the entire population in the 16S rDNA data (WGS-based estimates also range ∼6%), and up to 26.8% in sample P11. This division was statistically enriched in diseased samples (p< = 0.05, Metastats [30], Figure 1). TM7 is a novel candidate bacterial division with no cultivated representatives, and previous studies have shown microbes from this division to be commonly found in the human oral flora but at relatively low abundance, generally around 1% of the population [31], [32], though abundances as high as 8% were previously reported [6]. The high abundance of TM7 microbes present in our samples, and their correlation with periodontal disease, indicate that the prevalence of this poorly studied bacterial division within the oral cavity, and its role in disease, have yet to be fully appreciated.

When comparing healthy and diseased samples we observe a shift in the composition of the oral microbiota (Figure 1 and Table S1), supporting the well characterized transition (*p* value<10^−15^ using Fisher’s exact test) from a gram-positive dominated community in the healthy samples, to a gram-negative dominated community in periodontal disease [12]. On one hand, our findings recapitulate prior results that indicate that the gram-negative genera *Selenomonas* [33], [34], *Prevotella* [35], *Treponema* [36], *Tannerella* [36], *Haemophilus* [37] and *Catonella* [38] are significantly enriched in periodontal disease. Further, we have found a set of gram-positive genera that are significantly enriched in healthy samples: *Streptococcus, Actinomyces,* and *Granulicatella*. Surprisingly, however, neither *Fusobacterium*, nor *Porphyromonas* were found to be significantly more abundant in the periodontal disease samples, despite being previously implicated in this disease [12], [39]. This is likely due to the high variance in the abundance of these organisms across our samples, as well as the small sample size which affects our statistical power.

Clustering analysis (Figure 1) reveals sample H31 (a control) to have a microbiota most similar to the diseased samples. This observation prompted a careful analysis of the clinical data collected during sampling. The data revealed some symptoms of mild periodontal disease (such as bleeding at probing time, see Materials and Methods for more details) that were not found in any of the other healthy samples, indicating that the microbiota may shift into a disease state before the full clinical symptoms of the disease are apparent. Also note that the diseased samples (including H31) cluster together tightly while the healthy samples are more widely distributed. This phenomenon is discussed in more detail below.

Taxonomic enrichment, however, cannot fully explain the etiology of periodontal disease. All organisms that exhibit an enrichment in either healthy or diseased samples are present in all the samples, irrespective of disease status, i.e. the mere presence of pathogens in the periodontal pocket is not sufficient to trigger periodontitis. The disease might be correlated with the presence of specific virulence factors within the genomes of particular pathogens, or might be initiated once the abundance of one or more pathogens crosses a specific threshold. The mechanisms that keep pathogenic bacteria ‘in check’ during health but allow them to bloom during disease are not yet understood. These observations support our suggestion that a full understanding of periodontal disease requires whole-genome and whole-system analyses.

### Metabolism, Virulence Factors and Drug and Metal Resistance as Disease Signatures

**Figure 2:**
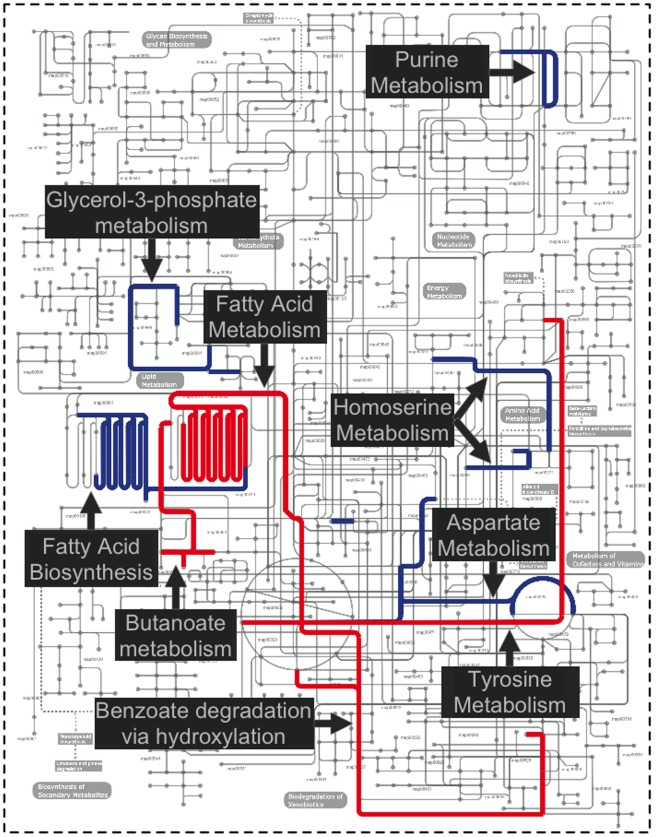
Metabolic pathways present in our samples. Dark blue – significantly enriched in healthy samples (p<0.05, MetaPath [84]); Dark red– significantly enriched in diseased samples (p<0.05, MetaPath). (Figure constructed with iPath [85]).

In addition to providing a taxonomic overview, our metagenomic sequencing data contain high-resolution functional information. We annotated the function of genes identified in the assembled whole-metagenome data according to the KEGG Orthology, and used the resulting data to compare the functional potential of the oral microbiome in health and disease. The metabolic profiles of healthy and diseased samples differ in a number of important ways (Figure 2). The diseased microbiome is enriched in metabolic functions that are consistent with a parasitic lifestyle made possible by the availability of nutrients derived from the degradation of host tissue and from bacterial cells destroyed by the host immune response (Table S2 for the statistical significance of enrichment of individual processes). Among these are functions for fatty acid metabolism and acetyl-coenzyme A degradation, aromatic amino acid degradation, ferrodoxin oxidation, and energy-coupling factor (ECF) class transporters. The periodontal pocket has been previously shown to be enriched for such nutrients in patients with periodontitis [40]. Several of these metabolic functions have also been associated with an intracellular lifestyle (e.g. fatty acid metabolism [41]), or with anaerobic metabolism (e.g. ferrodoxin oxidation, and acetyl-CoA degradation), highlighting the diversity of survival strategies employed by the microbes inhabiting the periodontal pocket during disease. Also enriched in disease are a number of virulence factors such as the presence of conjugative transposons, type IV secretion systems, and the biosynthesis of toxic factors (e.g. acetone, butanol, and ethanol biosynthesis), as well as the Lipid-A of lipopolysaccharide (LPS) biosynthesis. LPS is a group of molecules known to trigger host immune response and inflammation and their enrichment in disease provides a possible explanation for the systemic impact of periodontitis on the human host.

Finally, the periodontal disease samples are enriched in a number of functions related to drug and metal resistance (mercury, cobalt-zinc-cadmium). Mercury resistance has been previously characterized as a common feature of oral bacteria, even in the absence of mercury-containing amalgam, and is frequently associated with antibiotic resistance [42]. The role drug resistance plays in disease is, however, unclear as antibiotic resistance factors are present in both healthy and diseased samples.

Comparatively, only a few pathways are significantly enriched in the healthy microbiome (or depleted in the diseased microbiome), including pathways for fatty acid biosynthesis, purine metabolism, and glycerol-3-phosphate metabolism. Certain fatty acids have been shown to have a protective role in periodontal health [43], [44], [45] and it is possible that some of these are synthesized by the healthy microbiota. However, most of what is known about the role of fatty acids in periodontal health is based on nutritional studies and the contribution of the oral microbiota has yet to be characterized. Glycerol-3-phosphate is a lipid metabolite that has been shown to occur in higher concentration in periodontal disease samples [46]. Our study hints that a possible explanation for this observation is a decrease in the ability of the disease microbiome to metabolize this compound. Also enriched are genes related to homoserine metabolism, possibly related to quorum sensing functions within the healthy microbiome, as homoserine lactones are frequently used as quorum sensing molecules in oral bacteria [47]. The enrichment, within our healthy samples, of the reactions downstream of homo-serine lactone pathway may indicate a fully functioning quorum sensing system, allowing for the communication between organisms that is the hallmark of a healthy biofilm system. In poly-microbial biofilms it has been shown that mutants lacking quorum-sensing molecules, while able to construct biofilms, are unable to obtain the correct structure and thickness [48], [49]. The depletion of pathways related to quorum sensing in our diseased samples may indicate a possible cause of disease progression due to the inability of the healthy microbiome to maintain a protective biofilm.

### A Systems Level Perspective on Oral Disease

**Figure 3:**
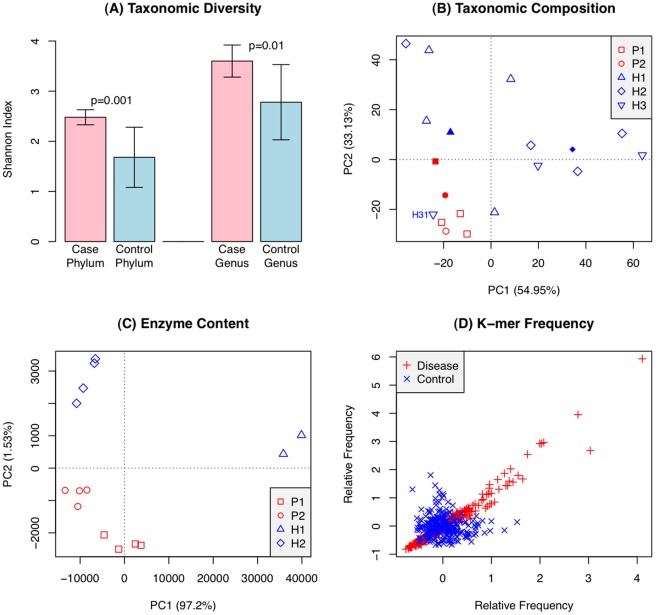
Systems-level analysis reveals the disease state to occupy a narrow region within the space of possible states for the microbiome. A – Shannon diversity calculated from 16S rDNA data is significantly higher in diseased samples (community is more diverse). B – Principal Component Analysis of the taxonomic compositions from 16S rDNA (empty symbols) and pooled WGS data (filled symbols). Disease samples cluster together in the bottom left corner. Sample H31 (tooth with incipient periodontal disease from an otherwise healthy patient) clusters together with the disease samples. C – Principal component analysis of the enzyme content of samples based on metagenomic sequencing. The PCA graph shows a tighter clustering of disease samples (red) relative to the healthy ones (blue). This suggests that the disease state may be linked to a specific metabolic configuration, and that the space of disease configurations is more constrained than the healthy one. Replicates (forward/reverse reads from one or two instrument lanes) are shown separately as identical symbols, and exhibit minimal metabolic variation within each sample. D – Comparison of relative frequencies of tetramers (4 bp motifs) in metagenomic reads across disease cases (in red, P1 vs. P2) and across control cases (in blue, H1 vs. H2). Based on the relative frequencies of tetramers, disease samples are more similar to each other (points lie along the diagonal) than controls are to each other.

The functional characterization reported above suggests that, beyond the taxonomic details, one can identify ecosystem-level signatures of periodontal disease consistent with its clinical manifestations. However, from the above analysis, it is still not clear whether these signatures reflect isolated instances of disease-related molecular processes, or fit into a coherent picture of the disease as a predictably different state of the whole oral microbial flora. We addressed this question by performing additional analyses at different levels of resolution, and found that a major systemic change seems to be identifiable between the healthy and diseased microbiomes. The diseased samples harbor a more diverse microbial community (as measured by the Shannon diversity index, Figure 3A), yet clustering analysis at the taxonomic level (Figure 1, Figure 3B) and in terms of enzyme content (Figure 3C), as well as pairwise comparisons of individual healthy and diseased samples based on tetramer (subsequences of length 4) frequencies (Figure 3D), all indicate that disease samples are more similar to each other than the healthy samples. In other words, the diseased state appears to be associated with a constrained and predictable region in the space of all possible states a microbiome can take. Thus, although the periodontal disease microbiomes are more diverse in terms of community structure, that structure is quite similar across different patients. In contrast, the healthy microbiome in any individual patient has relatively lower taxonomic diversity, but its exact composition differs significantly across patients.

**Figure 4:**
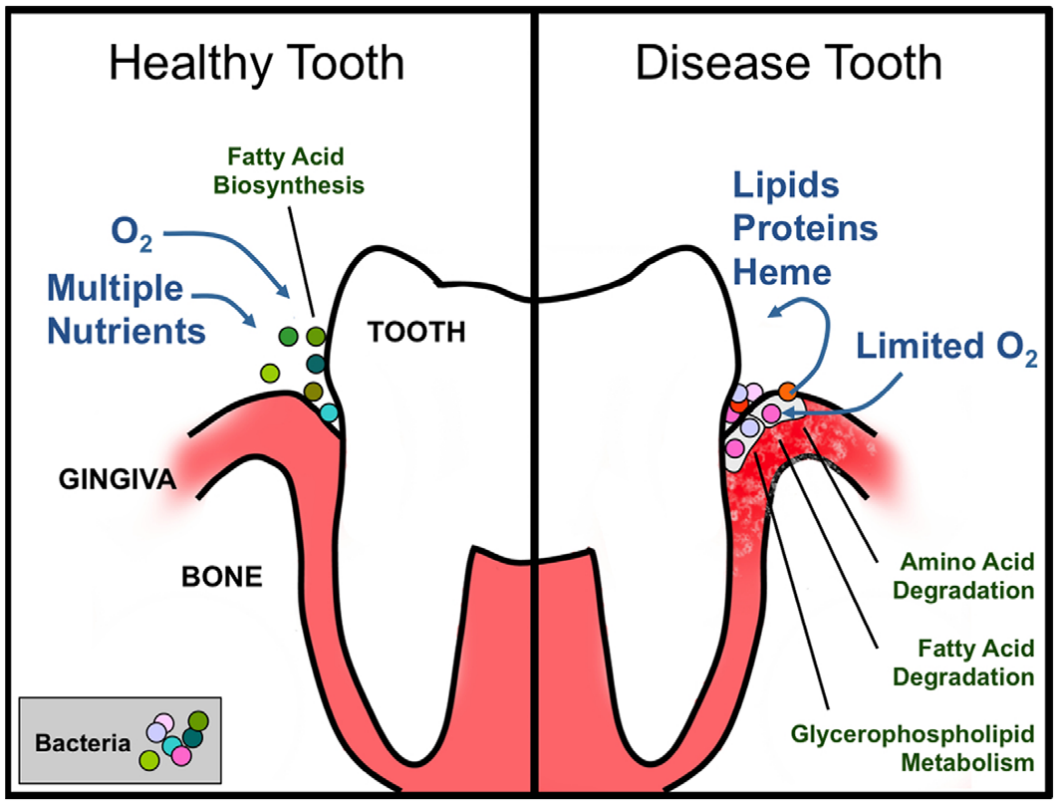
Schematic representation of the putative metabolic lifestyle shifts associated with the change in microbial flora around the tooth and gum tissue upon the transition from a healthy (A) to an advanced periodontal disease (B) state. The healthy state is dominated by the bacterial genera *Streptococcus, Fusobacterium, Actinomyces,* and *Corynebacterium*, whereas the disease state is primarily dominated by pathogenic genera such as *Prevotella, Leptotrichia, Treponema,* and *Fusobacterium*.

Combined with the metabolic analyses described above, these results suggest that some systems-level changes may be associated with periodontal disease and the transition between health and disease. Microbial consortia in healthy individuals (Figure 4A) may rely on a highly diverse and rapidly changing supply of nutrients, as well as on good availability of oxygen for respiration. The relative paucity of enriched pathways in our healthy case analysis may reflect the diversity of metabolic pathways represented in the community. This is also supported by the clustering analysis of 16S rDNA data (Figure 3B) and of enzyme frequency data (Figure 3C), which show that the healthy data points do not tend to cluster together (Figure 4A, bottom left inset), and is consistent with a community with a lower taxonomic diversity (Figure 3A). On the contrary, the metabolic functions present in the microbial flora associated with periodontal disease (Figure 4B) seem to display a significant enrichment in specific metabolic pathways, compatible with an oxygen poor environment [50], and the availability of amino acids and lipids as major carbon sources. This may reflect the invasion of microbial pathogens (e.g. *Prevotella intermedia* which is enriched in the diseased samples) into human cells (both epithelial cells and macrophages). The disease flora is rich in lipid degradation pathways, as well as other known virulence-related activities, such as LPS biosynthesis. In turn, the consistency of the intracellular environment across different patients may explain why the disease points tend to cluster together in the Principal Component Analysis (PCA) plots. The ensuing picture is that the disease state is an attractor in the space of metabolic functions, with enrichment in cytotoxic and parasitic functions.

### De Novo Assembly of Oral Microbes

The analyses we presented above have focused either exclusively on organisms (16S rDNA diversity) or biological function (metabolic analysis), thus ignoring the important link between organisms and the functions they perform. This connection can only be made by reconstructing partial or entire organisms from the community through metagenomic assembly. Currently, no practical genome assemblers exist that are specifically designed for large-scale metagenomic assembly, thus we relied on a hybrid assembly approach that combined *de novo* assembly using SOAPdenovo [51] (assembler used in a recent metagenomic analysis of gut microbes [52]), and alignments against a collection of oral microbes (Methods). The results shown in Table 2 demonstrate the power of this hybrid approach, which leads to an average of 4.4 and 2.1 times larger (in terms of N50 contig size) assemblies than *de novo* assembly and comparative assembly, respectively. Despite the relatively low level of coverage in our data, we obtain fairly contiguous assemblies (average N50 contig size of 3.5 Kbp), and are able to assemble up to about 50% of the total number of reads in our data-set. Furthermore, consistent with our previous observation that the periodontal disease samples are more diverse, the corresponding assemblies are also more fragmented (average N50 contig size is 1.2 Kbp in diseased samples versus 5.8 Kbp in healthy samples). In addition, a pooled assembly of all four samples results in dramatically increased contig sizes (max contig size is 16.9 Kbp in pooled assembly versus 7.6 Kbp in individual assemblies), indicating these samples contain closely related organisms.

**Table 2:** Assembly statistics of metagenomic shotgun reads for contigs that are > = 300 bp using (1) SOAPdenovo, (2) comparative assembly and (3) a hybrid approach that uses MINIMUS to combine the contigs from the previous two methods.

Sample		Assembly approach	# Contigs	Length (Mbp)	Max (Kbp)	N50 (bp)	N90 (bp)	Reads assembled		
								# (M)	%	
**P1**		SOAPdenovo	22,226	11.8	12.0	583	368	1.2	12.45	
		Comparative	26,464	16.7	16.0	1113	598	1.3	13.21	
		Hybrid	37,213	24.6	16.0	1829	1025	2.3	23.42	
**P2**		SOAPdenovo	12,966	6.3	3.3	352	0	6.7	14.23	
		Comparative	13,841	8.5	35.2	490	0	5.7	11.69	
		Hybrid	21,835	12.5	37.6	647	396	10.5	21.39	
**H1**		SOAPdenovo	45,658	3.1	22.6	3042	1648	5.0	40.20	
		Comparative	46,036	3.3	18.6	2437	1559	3.5	28.21	
		Hybrid	63,688	5.1	19.0	7567	3953	6.7	53.18	
**H2**		SOAPdenovo	18,048	10.6	12.7	616	352	1.7	25.51	
		Comparative	16,107	13.6	26.8	1543	689	2.2	32.33	
		Hybrid	20,339	17.6	110.0	3934	1099	3.1	45.88	
**Pool**		SOAPdenovo	98,051	54.9	15.7	2035	1342	8.1	24.12	
		Comparative	63,506	60.1	44.6	8415	5474	8.4	24.89	
		Hybrid	115,718	93.4	229.8	16896	9245	13.4	39.87	

‘Pool’ represents the assembly of all four samples together. N50 or N90 is defined as the contig length such that equal or longer contigs produce 50% or 90% of 10 Mbp.

### Assembly of a TM7 Genome

As described above, we detected a higher presence of TM7 organisms in our samples than previously reported in literature. TM7 is a novel candidate bacterial phylum without cultivated species, and previous studies have shown its high prevalence in human oral flora but with very low abundances [31], [32]. The first sequence of a TM7 organism (TM7a) was generated through single-cell isolation in a microfluidic device, followed by whole genome amplification [53]. Due to the artifacts of the whole genome amplification approach, the resulting assembly is fairly fragmented (row 1 in Table 3). Here we relied on a hybrid assembly approach to reconstruct a more complete version of this genome, using the corresponding shotgun sequences generated in our project. Briefly, we started with the pooled assembly of all our samples and extracted all contigs that are mapped to the previously sequenced TM7a genome, and scaffolded these contigs using Bambus 2 [54]. Finally, we merged our TM7 assembly with the previously published assembly, derived from single-cell sequencing, in order to construct the most complete (to date) assembly of an organism from the TM7 group. The final assembly is still highly fragmented, comprising over 1,500 contigs (Table 3), however it contains almost 50% more sequence than the single-cell derived assembly (2.3 Mbp versus 1.7 Mbp), and the N50 contig size is two times larger (790 bp versus 389 bp). These results highlight the power of combining single-cell and metagenomic approaches when reconstructing the genomes of unculturable organisms from metagenomic samples (Figure S1 for the distribution of contig sizes).

**Table 3:** Assembly statistics (calculated on contigs > = 300 bp) for HOMD TM7a reference sequences (row 1), hybrid assembly from metagenomic shotgun reads (row 2), Bambus scaffolding of hybrid assembly (row 3), assembly from combining hybrid assembly and the HOMD reference sequences (row 4), and Bambus scaffolding of ‘combined reference’ (row 5).

Assembly	# Contigs	Total Length	Max Contig	3Mbp	
				N25	N50
**HOMD TM7a reference genome**	1,780	1.7Mbp	17.5 Kbp	1.9 Kbp	0.4 Kbp
**Hybrid assembly**	1,340	1.5Mbp	13.9 Kbp	1.8 Kbp	NA
**Scaffolds**	874	1.6Mbp	20.9 Kbp	5.1K bp	0.5 Kbp
**Combine reference**	2,222	2.2Mbp	17.5 Kbp	2.9 Kbp	0.8 Kbp
**Scaffolds**	1,593	2.3Mbp	33.7 Kbp	7.2 Kbp	1.8 Kbp

The N25 and N50 are calculated assuming a 3 Mbp genome size.

In addition, this improved TM7 genome assembly allows us to identify 703 genes that were not present in the original assembly (Methods for details). In order to evaluate the additional information contained in these genes, we annotated them using the COMBREX [55] system (Table S4). The analysis revealed several potential virulence genes including an EmrB/QacA family drug resistance transporter gene (Gene ID: 681_1) and two phage proteins (Gene IDs: 386_2 and 1828_4). These genes are not necessarily omissions from the original assembly, rather they could represent *de novo* insertions into the TM7 genome present in our sample. The set of ‘novel’ TM7 genes does, however, included several housekeeping genes (e.g., 10 ribosomal protein genes not present in the original assembly) which should be conserved across TM7 genomes, thereby indicating that our assembly improves upon our current understanding of the structure of the TM7 genome in addition to revealing strain-specific genomic variants.

### Genomic Variation in Actinomyces Naeslundii

**Figure 5:**
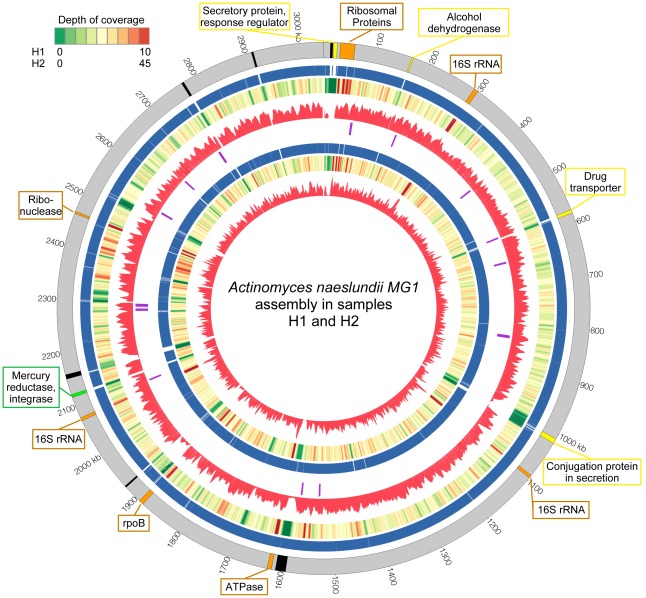
Comparative analysis of the *Actinomyces naeslundii* MG1 reference genome (HOMD SeqID SEQF1063), and assemblies from samples H1 and H2. Counting from outside, the first ring is the reference genome with genes annotated by colored bands: orange bands are conserved genes; green bands are genes involved in horizontal gene transfer; yellow bands are genes with known functions; black bands are genes with unknown functions. Only regions that are associated with genomic variations are colored and annotated. The second and sixth rings are the assembled contigs from sample H2 and H1. The heatmaps in the third and seventh rings represent the depth of coverage of the contigs with 5K bp window and 100 bp step for sample H2 and H1. The histograms in the fourth and eighth rings represent the scaled SNP rate with 5 Kbp window and 100 bp step for sample H2 (max = 0.08) and H1 (max = 0.03). The purple bands in the fifth ring represent regions with significantly higher theta values than average (p< = 0.05). Image generated with Circos [86].

Close analysis of one of the most abundant organisms in our samples (present at 24- and 6-fold coverage in samples H2 and H1, respectively, Table 4), a relative of *Actinomyces naeslundii* MG1 (sequence ID SEQF1063 in the Human Oral Microbiome Database (HOMD) database), provides evidence for structural variations distinguishing this strain from the reference strain originally isolated from a patient with mild gingivitis [56]. The average similarity between the assembled metagenomic contigs from our project and the reference sequence is 96.2% and 95.2% for samples H1 and H2, respectively (second and sixth ring in Figure 5). A number of genomic deletions with respect to the reference strain are apparent in our samples, several of which contain potential virulence factors. These differences could be explained by the fact that the reference genome was isolated from a patient with gingivitis, while in our samples the *Actinomyces* strains are predominantly associated with healthy samples. Most striking is a deletion at 2120 kbp containing a putative mobile element encoding a mercury resistance locus (including a mercury resistance gene, a site-specific recombinase, and an integrase). Mercury resistance is commonly found in oral bacteria, frequently associated with antibiotic resistance [42]. Interestingly, gene set enrichment analysis of the entire metagenomic data-set reveals an enrichment of mercury resistance genes in the diseased samples (Table S2), possibly due to the association of these genes with virulence loci. Several other deletions also appear to encode virulence factors - a drug transporter (at position 580 kbp in the reference strain) and an alcohol dehydrogenase gene (at position 165 kbp) – further underscoring the difference between the pathogenic reference strain and the presumably commensal *Actinomyces* strains found in our samples. Another two deletions (at positions 20 kbp, and 1010 kbp) contain genes predicted to encode proteins involved in secretion and response regulation. These deletions occur at slightly different locations in the two samples we analyzed, suggesting they may be subject to rapid evolution.

**Table 4:** Assembly statistics (calculated on contigs > = 100 bp) for *Actinomyces naeslundii* MG1 in sample H1 and H2.

Reference genome	Length	Sample	# Contigs	Total length	Depth of coverage	Max Contig	N50	N90
*Actinomyces naeslundii*MG1	∼3Mbp	**H1**	2850	∼2.8Mbp	6.2	14.5 Kbp	1.7 Kbp	0.1 Kbp
		**H2**	1168	∼2.9Mbp	24.3	28.6 Kbp	5.5 Kbp	0.9 Kbp

The N50 and N95 are calculated based on the MG1 genome size.

Further evidence of the adaptation of *Actinomyces* to the oral environment is revealed by the analysis of single nucleotide polymorphism (SNP) densities. In Figure 5 (rings 6 and 8) we highlight the regions of the genome that have higher than expected SNP densities (>2 standard deviations from the mean). The most polymorphic regions (Table S3) correspond to genes predicted to be involved in the adaptation of an organism to its environment: transcriptional regulators, known to evolve rapidly in bacteria [57], [58], and ABC transporters [59]. Another highly polymorphic region occurs within the glyceraldehyde-3-phosphate dehydrogenase (GAPDH) gene, a virulence-associated protein originally identified in Streptococci, which plays an important role in the colonization of periodontal pockets by interacting with plaque-forming bacteria [60]. GAPDH was also shown to mediate the interactions between Streptococci and *Porphyromonas gingivalis* fimbriae [61], [62], possibly contributing to the colonization of the subgingival pocket by *P. gingivalis*. These observations are consistent with previous findings of high-SNP densities within genomic regions surrounding recombination events [63].

### Conclusions

Our study represents a novel step towards characterizing the genomic composition of the microbial communities associated with periodontal disease. We have demonstrated that the subgingival microbiome can be effectively interrogated through high-throughput sequencing, and that the resulting data provide valuable insights into the molecular underpinnings of periodontal disease.

Despite a relatively small amount of bacterial sequence data recovered from our samples (primarily due to the high level of human DNA contamination), a combination of comparative and *de novo* assembly approaches was able to reconstruct large genomic segments from several dominant organisms in our samples, thereby allowing a better reconstruction of an unculturable TM7 organism (in conjunction with data generated through single cell genomic approaches), and providing a glimpse at the genomic variation (and possible association with virulence) within *Actinomyces* genomes. Better assemblies were possible in samples that were sequenced more deeply (e.g., sample H1), indicating the need to sequence the oral environment more deeply than has been done in this study. Furthermore, assembly quality roughly correlated with disease status, partly confirming our observation (based on 16S rDNA data) that diseased samples had a higher microbial diversity. This observation also highlights a limitation of existing assembly tools in dealing with genomic diversity, further underscoring the need for the development of metagenomic-specific genome assemblers.

The analysis of the TM7 and *Actinomyces* genomes revealed signatures consistent with recombination events possibly associated with virulence factors. Lateral transfer of virulence determinants through phages and recombination is well documented in the bacterial world, leading to a partial separation between function and phylogeny, thus, suggesting the need for metagenomic and functional analyses as a complement to taxonomic surveys of host-associated microbiota.

Taxonomic analyses of the data we generated are consistent with a well-established community shift from a gram-positive dominated healthy microbiome to a gram-negative dominated diseased microbiome, which is also enriched in a number of oral pathogens. The molecular mechanisms that underlie and cause this transition are, however, unknown. Here we have shown that functional information derived from whole-metagenomic data provides a valuable complement to the taxonomic data and allows us to develop a novel theory of periodontal disease. The healthy state is highly regulated by the host immune system and interactions between community members to maintain a community dominated by few “good” microbes, usually gram-positive Actinobacteria or Streptococci. The transition to periodontal disease involves a disruption of the host-microbiome interactions that results in a more even community structure composed by a broad range of organisms that can thrive in the oral environment. The presence of pathogens within this community can lead to the clinical manifestations of periodontal disease, which in turn can lead to additional changes in the community due to the increased availability of nutrients released by the damaged tissue. As a result, the periodontal disease microbiome eventually settles into a state characterized by a diverse population of microbes adapted to a parasitic lifestyle made possible by the disrupted host homeostasis. One of the samples from our study was characterized by a microbiota typical of a diseased state, yet the corresponding tooth was just starting to show some of the clinical symptoms of disease. This observation implies that dysbiosis precedes the clinical manifestation of disease, and that the oral microbiota could be a potential tool for the early diagnosis of periodontitis.

The large variability we observe between healthy samples, and even between different teeth of a same person, highlights the limitation of using data derived from cross-sectional studies to define what the core “normal microbiome” means. Furthermore, case-control studies are likely insufficient to determine the causal agents of periodontal disease – the organisms found to dominate the diseased microbiome (the “usual suspects” commonly described in the literature) may simply be a symptom of the disrupted subgingival environment rather than the primary cause of disease. The “usual suspects” approach considers presence and absence of specific bacteria to be the critical precondition for disease, however, our data support a more nuanced approach that considers quantitative and genomic differences as the critical factors when moving from health to a diseased state. Longitudinal studies are necessary to characterize the dynamic changes that occur in the oral microbiome in response to environmental changes (food intake, changes in the host, etc.) and to track the transition between the healthy and diseased states, and the return to health after treatment.

It is important to note that the analyses described above are a preliminary pilot project with limited sample size, and our observations must be confirmed in more extensive studies. Furthermore, we focus on whether the microbiome has the potential to perform certain biological functions, and on determining the relative fraction of the microbial population that can perform a particular function. These results (as well as those of similar metagenomic projects) must be complemented by experimental studies aimed at determining whether the biological processes statistically enriched in disease are actually active in the subgingival pocket.

As others have previously reported, and as observed in the data we have shown here, periodontal disease is the result of a disruption of the complex interactions occurring within the subgingival microbiome and between the microbiome and the host. A full understanding of the etiology of periodontal disease will only be possible through further in-depth systems-level analyses of the host-microbiome interaction.

## Materials and Methods

### Subject Population

The subject population consisted of 5 patients who were in good general health and were recruited between August and November 2009 at the Clinical Research Center, Boston University Goldman School of Dental Medicine. Written informed consent was obtained from all enrolled individuals. The study protocol was reviewed and approved by the Institutional Review Board at the Boston University Medical Center. All subjects had at least 12 natural teeth with >20 years of age (age range, 28–45 years). Subjects diagnosed with chronic periodontitis (n = 2) were selected among those who had at least six sites with probing depth ≥6 mm and attachment loss ≥5 mm. Subjects in the control (periodontally healthy) group had no pockets >3 mm and no attachment loss >2 mm at any site with no signs of periodontal inflammation characterized by bleeding on probing, redness, edema, and attachment loss, with the exception of subject 5 where one of the teeth (# 3, sample H31) exhibited mild bleeding at probing time consistent with initial periodontal disease. Sites with gingivitis were characterized by bleeding on probing, redness, edema, but no attachment loss (pocket depth ≤4 mm). Sites from chronic periodontitis subjects further characterized as mild, moderate or advanced periodontitis sites based on the pocket depth. Mild periodontitis was characterized with pockets >4 mm but not more than 5 mm; moderate periodontitis was characterized with pockets >5 but <7 mm while advanced periodontitis was characterized with pockets >7 mm. Healthy group consisted of subjects of Asian, Caucasian and African American origin, while periodontitis subjects were of Caucasian and African-American origin. Exclusion criteria included pregnancy, lactation, systemic conditions that could affect the progression or treatment of periodontal diseases. In addition, none of the subjects had received systemic antibiotics or periodontal therapy in the previous 6 months.

### Subgingival Plaque Sampling and Isolation of Bacterial DNA

After the removal of supragingival plaque with sterile gauze, individual subgingival plaque samples were taken from the mesio-buccal aspect of four molar teeth in four quadrants (upper right, upper left, lower right and lower left) per subject using sterile periodontal curettes (Hu-Friedy, Chicago, IL). Each sample was placed in a separate sterile 1.5-ml tube containing 200 µl TE buffer (50 mM Tris-HCl, 1 mM EDTA; pH 7.6). Bacterial DNA extraction was performed using commercially available DNA purification kit (Epicentre MasterPure™, Madison, WI) according to manufacturer’s guidelines. First, the debris was separated by centrifugation at 4°C and supernatant was transferred to another microcentrifuge tube and pellet was discarded. The collected supernatant was mixed with 500 µl of isopropanol and centrifuged at 4°C for DNA isolation. Isopropanol solution was carefully removed without dislodging the DNA pellet. The DNA pellet was rinsed with 75% ethanol and residual ethanol was removed. The sample was resuspended in 25 µl of TE buffer and stored at −80°C until analysis.

### 16S rDNA Sequencing Protocol

DNA samples were amplified using the primers: 5′-GCCTCCCTCGCGCCATCAGacacactgCATGCTGCCTCCCGTAGGAGT and 5′-CCTATCCCCTGTGTGCCTTGGCAGTCTCAGAGAGTTTGATCCTGGCTCAG to initiate the reaction. The underlined portions of the primers corresponded to ‘universal’ bacterial primers 338R and 27F primer, the small letters contained a barcode specific to each well and the 5′ end of the primer was specific to the 454 specific protocols. Each reaction had 5 units of Choice DNA Taq polymerase (Denville Scientific), 100 uM of dNTPs, 1× reaction buffer and 2 mM MgCl_2_ and occurred for 30 cycles of 94°C for 30 sec, 50°C for 30 sec, and 72°C for 30 sec. The presence of amplified products was confirmed by gel electrophoresis. Approximately equimolar amounts of product were pooled and gel purified. Sequencing was performed using the Lib-L kit following instructions from the manufacturer (Roche).

### Data Collection, Sequencing and Preprocessing

16S rDNA sequences were processed and filtered based on quality with an in-house pipeline as follows. First, sequences containing at least one unrecognizable base-pair (‘N’), and that were too short (<75 cycles of the 454 instrument) were excluded from further analysis. Then, barcode sequences were deconvoluted and removed.

Metagenomic sequencing was performed on pooled DNA from multiple teeth in order to obtain sufficient DNA concentrations for library construction. Metagenomic shotgun sequences were obtained from the Illumina instruments in fastq format and were trimmed for quality using the FastX Toolkit (Hannon Lab, CSHL) with the following parameters: (1) minimum length 25, and (2) q-value cutoff 20. Sequences containing at least one ambiguity character (‘N’) were also removed. The remaining sequences that passed the quality trimming outlined above were mapped to the human genome reference (NCBI build 37 v 1) downloaded from NCBI using Bowtie with parameters (−v 3; at most 3 mismatches) If one of the sequences from a paired end matched the human genome, then both sequences were removed from the data-set. The remaining reads were mapped against the human sequences in the NCBI *nr* database using Basic Local Alignment Search Tool (BLAST) in order to remove human sequences not present in the NCBI human genome reference. For this additional check we required at least 95% global identity (since BLAST is a local alignment algorithm, our calculation also takes into account the length of the unaligned segments flanking the ‘hit’ reported by BLAST).

### Comparative Assembly

We mapped and assembled the samples against reference sequences for oral microbes extracted from the Human Oral Microbiome Database (HOMD, http://www.homd.org) [64] as follows:

1. We used MUMmer [65] (-maxmatch -l 20 -b) to map the individual reads against the HOMD reference database.
2. Reads that mapped with higher than 80% global identity were then assembled based on the mapped coordinates of the reads.
3. This process was repeated using a 90% similarity threshold, but mapping the reads against the assemblies generated at step 2, rather than against the HOMD database.
4. The resulting contigs were then combined with the results of a de novo assembly of the data, as described in more detail below.

All customized scripts used to run this analysis can be obtained by request from the authors and will soon be released as part of an open-source package for comparative assembly of metagenomic data [Liu et al. in preparation].

### De Novo Assembly Using SOAPdenovo

We used SOAPdenovo V. 1.04 [66] with parameters −K 23 and −M 3, as previously used by the MetaHit project [52] to assemble gut microbiome data.

### Combining Comparative and de Novo Assembly Data

Contig sequences longer than 100 bp, which were generated by our customized comparative assembly pipeline (workflow presented above) and by SOAPdenovo, were combined and assembled using MINIMUS [67] with the following parameters: (1) minimum overlap length 40 bp, and (2) overlap error rate is 0.1.

### Assembly and Gene Prediction of TM7 Genome

The reference genome of TM7a was downloaded from HOMD (http://www.homd.org/). We pooled all available metagenomic sequences together, and performed comparative assemblies against the existing TM7a reference sequences as described above. The resulting contigs were combined together with the *de novo* assemblies of all metagenomic sequences using MINIMUS, resulting in a hybrid assembly for TM7a. The resulting assembly was then further combined with the HOMD TM7a reference genomic sequence. After assembly, scaffolds were created using BAMBUS 2 using the available mate-pair information.

We used MetaGene [68] to predict genes (> = 300 bp) in the TM7 reference genome (NCBI accession NZ_ABBV00000000) as well as in our combined assembly described above. We, then mapped (using BLASTN) the predicted genes from our assembly against the predicted genes from the TM7 reference genome. A match was defined as E-value< = 0.00001, % similarity > = 90%, and more than 50% of the query sequence is aligned. Genes found in our assembly that did not match any gene in the reference strain were considered novel and subjected to functional annotation using COMBREX.

### Estimation of SNP Rates and Genetic Diversity

After assembly, the shotgun reads were mapped back to the contigs using Bowtie [69] allowing at most 3 mismatches. To avoid sequencing and mapping errors we used a conservative approach as suggested in [70]: we only retained SNPs occurring in regions with a depth of coverage higher than 4, and with each individual haplotype represented in at least two different reads. The SNP rate was calculated using a 5 Kbp window and a 100 bp step size.

We adapted the approach used in [71], [72] to infer the genetic diversity θ from metagenomic shotgun sequencing data using composite likelihood estimators while accounting for a constant sequencing error rate. First we classified the nucleotide positions of the assembled contigs into *k* groups, where *k* is the maximum depth of coverage of the contigs, and positions within the same group have the same depth of coverage. The number of nucleotide positions in each group is denoted by *n*_1_, *n*_2_, …, *n*_k_. Considering the large number of bacteria in the sampled community relative to the number of reads sequenced, the probability that each read derives from a different individual microorganism is close to one. Thus, we have a population size equal to the depth of coverage at every site in the assembled contigs [71]. Consequently, the estimator can be obtained by calculating the expected number of true SNPs and false SNPs due to sequencing errors [72]. Then for a particular nucleotide group with the same depth *d*, assuming an infinite sites model, the expected number of segregating sites is 
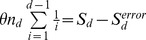
, where *S_d_* is the observed number of segregating sites (SNPs) from data and 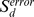 is the expected number of segregating sites induced by sequencing errors. For a nucleotide position with depth of coverage *d*, the probability of at least two mutations (*x> = 2*; considered as true SNPs) induced by sequencing error (*e*) is:
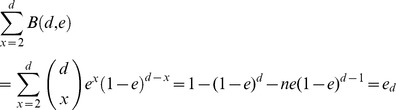
Since there are *n_d_* such sites with depth of *d*, the expected number of segregating sites induced by error is 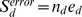. Hence the estimated 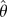 for regions with *d* depth of coverage is 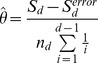


Finally, summing over all groups we get the equation.
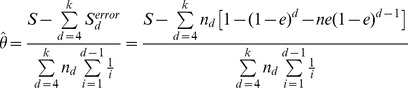
In our calculation, we assume a constant sequencing error rate *e = 0.01*. The θ value is calculated using a 1 Kbp window moving average (which is roughly the average gene size in bacteria) with 100 bp step size.

Regions of the genome that had a value of θ more than 2 standard deviations higher than the average were flagged as potential polymorphism hotspots (Table S3).

### Clustering and Annotation of 16S rDNA Sequences

The entire set of trimmed 16S rDNA sequences were clustered into Operational Taxonomic Units (OTUs) with the program DNACLUST [73], using a 1% radius (−r 2). To obtain the taxonomic identities, the OTU centers were aligned using BLAST to the RDP database [74] augmented with oral clones from the HOMD database [64], and were annotated using the lowest common ancestor approach (similar to the approach in [75]). The assignment process is conservative: (1) only sequence with at least global 98% identity with the reference is classified; (2) if there are more than one equally good best hits, then the sequence is classified using the lowest common ancestor approach; (3) otherwise it is classified as unknown. Finally, the taxonomic label of the OTU center is transferred to the sequences from the same OTU cluster. The resulting data was organized in a collection of tables at different taxonomic levels containing each taxonomic group as a row and each sample as a column. These tables formed the substrate for the further statistical analyses.

### Antibiotic Resistance Genes Annotation

Shotgun reads are annotated against the Antibiotic Resistance Genes Database (ARDB) reference genes [76] using BLASTX with the following thresholds: (1) at least 60 bp long high-scoring segment pairs, and (2) 90% or 95% similarity cutoff at the amino acid level.

### Seed Functional Mapping

Sequences that were preprocessed for removal human contamination were uploaded to the MG-RAST online webtool (version 2.0 [77] metagenomics.anl.gov/v2). Results were downloaded and parsed into individual files, one per patient, using PERL. Only annotations with a confidence of 1E-5 or lower were kept for further processing resulting in 1,130,510 annotations (representing 75,742 distinct functional labels). In an attempt to correct for under-counting of low-abundance sequences, we used the Laplace correction and added one to all counts.

### GSEA

To perform the gene set enrichment analysis (GSEA), we used the gene set enrichment analysis (GSEA) tool version 2.07 [78], downloaded from the Broad Institute website (www.broadinstitute.org/gsea/index.jsp). GSEA was used to identify functional categories that were enriched in disease or control patients. Functional sub-classes from SEED (Version 12) [79] were used as the gene sets (total of 669). We added two gene sets which were not represented in SEED, ‘transposases’, and ‘transposon related’, bringing the total to 671). These new gene sets were created by searching for the title term (i.e. ‘transposases’, or ‘transposon related’) in the functional descriptions of the full set of SEED functional categories. We used the default parameter settings of the GSEA software, with two exceptions. In our analysis, to rank the functional classes, we used a ‘ratio of classes’ rather than the ‘signal to noise’ metric, and for the significance testing we permuted the gene set instead of the phenotype. We selected and reported all gene sets that had a p-value less than 0.005, and with a false discovery rate q-value cut off of 0.01.

### Calculating Tetramer Frequencies

K-mer frequencies, especially tetranucleotide frequencies in prokaryotic genomes, have been shown to carry a phylogenetic signal [80]. For each patient, we counted the occurrence of each sequence motif of length 4 for all the pre-processed sequence reads using a sliding window approach. The relative frequency of each motif was calculated as the number of occurrences of a specific motif (tetramer), divided by the total number of motifs of length 4. The number of expected motifs of length k based on the expectation from motifs of length k-1 was estimated using a Markov chain [81]. For example, the probability of observing a tetramer, such as “CTAG” is estimated from the transition probability of observing a “CTA” motif adjacent to a “TAG” motif at “TA” dinucleotides, or p(CTAG|CTA,TAG)  =  p(CTA) * p(TAG)/p(TA). The observed motif frequencies were normalized by subtracting the number of expected motifs from the number of observed motifs, and dividing the result by the number of expected motifs. Using these normalized values, we can visualize the tetramer frequency counts directly and compare them across samples. The tighter, linear grouping of the disease samples suggests that the corresponding communities are composed of organisms phylogenetically less diverse, compared to the larger, more diverse cluster of healthy samples.

### Taxonomic Diversity Analysis

Relative abundance estimates obtained from the 16S rDNA sequencing data were used to compute the entropy (Shannon diversity) for each of the 15 samples for which 16S rDNA data were generated. Separate analyses were performed at the genus and phylum levels, and the results were aggregated across clinical status. The statistical significance of the observed differences was estimated using a standard t-test.

### Taxonomy Based PCA

The taxonomic composition of the samples was estimated based on both 16S rDNA data (Methods above) and WGS data using MetaPhyler [82]. Each sample was represented as a vector of relative abundances of individual phyla, and the resulting vectors were subjected to Principal Component Analysis using the *princomp* function in R. Note that samples represented in both the 16S rDNA and the WGS data are represented twice in this analysis.

### Enzyme Based PCA

After pre-processing, metagenomic sequences were annotated with specific Kyoto Encyclopedia of Genes and Genomes (KEGG) orthology (KO) codes using BLAST searches against the KEGG sequence repository [83]. The KO numbers for each protein were mapped to EC numbers, using a combination of custom Perl scripts and a conversion table. The table was generated from data available from the KEGG ftp server (ftp://ftp.genome.jp/pub/kegg/). An E-value cut-off of 1E-05 was applied to the resulting annotations to remove non-specific BLAST hits. For each sample, we constructed a count matrix containing the number of reads mapping to each enzyme. We carried out a principal component analysis on this matrix, using the ‘princomp’ function in Matlab implemented with the default parameters. We then displayed the sample position on the first and second principal components in Figure 3C to visualize each sample’s relative distance.

## Supporting Information

Figure S1 **Histogram distribution of contig sizes.** The upper plot shows the distribution of contig size from TM7 reference genome, which is assembled from single-cell sequencing. The lower plot shows the distribution of contig size from the assembly that combines the contigs from TM7 reference genome and metagenome. Contig sizes that are > = 5000 bp are plotted as 5000 bp.
(TIF)

Table S1 **Differential abundance of genera between cases and controls.** P-values were computed with Metastats [30].
(PDF)

Table S2 **Summary of microbial functions enriched in diseased or control samples.** CLINICAL – indicates whether enrichment occurs in disease or control samples; METHOD – method used to compute significance of enrichment; SIGNIFICANCE – Method-specific assessment of the significance of enrichment: for GSEA [87] we report both the p-value and the q-value obtained by correcting for the False Discovery Rate (FDR); for MetaPath [84] we report both the raw p-value for enrichment (p-abund) and p-value corrected for the structure of the metabolic network (p-struct).
(PDF)

Table S3 **Genomic regions with nucleotide diversity θ values that are more than two standard deviations away from the mean.**
(PDF)

Table S4 **COMBREX** [**55**] **functional annotations of new genes predicted from TM7a metagenomic assembly.**
(PDF)
