# Supplementary figures and images for: Deep Sequencing of the Oral Microbiome Reveals Signatures of Periodontal Disease
Source: PLoS One. 2012 Jun 4;7(6):e37919. doi: 10.1371/journal.pone.0037919 (PMC3366996; doi:10.1371/journal.pone.0037919)

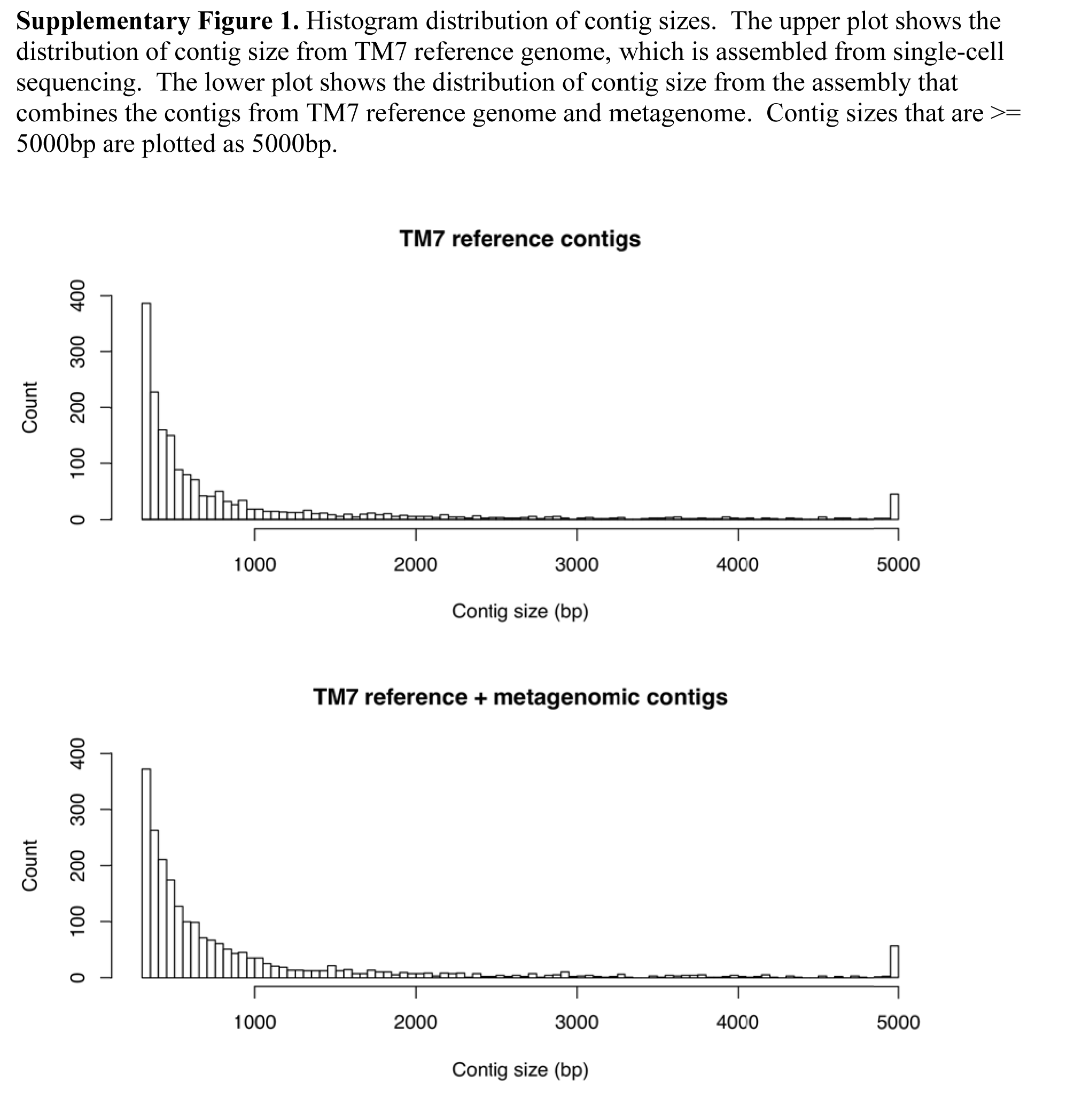

Supplement: Figure S1 — Histogram distribution of contig sizes. The upper plot shows the distribution of contig size from TM7 reference genome, which is assembled from single-cell sequencing. The lower plot shows the distribution of contig size from the assembly that combines the contigs from TM7 reference genome and metagenome. Contig sizes that are > = 5000 bp are plotted as 5000 bp. (TIF) [file pone.0037919.s001.tif]
