# Supplementary material for: Deep Sequencing of the Oral Microbiome Reveals Signatures of Periodontal Disease
Source: PLoS One. 2012 Jun 4;7(6):e37919. doi: 10.1371/journal.pone.0037919 (PMC3366996; doi:10.1371/journal.pone.0037919)
Supplement: Table S1 — Differential abundance of genera between cases and controls. P-values were computed with Metastats [30]. (PDF) [file pone.0037919.s002.pdf]

Supplementary Table 1. Differential abundance of genera between cases and controls.
P-values were computed with Metastats [1]

Genus	*p* value (Metastats)	Case		Control	
		Mean	SEM	Mean	SEM
Selenomonas	<0.01	3.54	0.47	0.61	0.21
Prevotella	<0.01	24.84	4.33	2.95	1.95
Tannerella	<0.01	0.89	0.26	0.02	0.01
Granulicatella	0.01	0.04	0.01	0.61	0.21
Actinomyces	0.01	1.70	0.23	19.06	6.55
Streptococcus	0.02	1.15	0.26	20.31	7.54
Catonella	0.02	0.72	0.28	0.04	0.02
Treponema	0.03	3.66	1.17	0.71	0.49
Haemophilus	0.04	0.05	0.02	1.90	0.83
Gemella	0.07	0.40	0.23	1.15	0.31
Fusobacterium	0.08	13.30	3.04	5.78	2.51
Filifactor	0.10	0.82	0.42	0.07	0.07
