# Supplementary material for: Deep Sequencing of the Oral Microbiome Reveals Signatures of Periodontal Disease
Source: PLoS One. 2012 Jun 4;7(6):e37919. doi: 10.1371/journal.pone.0037919 (PMC3366996; doi:10.1371/journal.pone.0037919)
Supplement: Table S2 — Summary of microbial functions enriched in diseased or control samples. CLINICAL – indicates whether enrichment occurs in disease or control samples; METHOD – method used to compute significance of enrichment; SIGNIFICANCE – Method-specific assessment of the significance of enrichment: for GSEA [87] we report both the p-value and the q-value obtained by correcting for the False Discovery Rate (FDR); for MetaPath [84] we report both the raw p-value for enrichment (p-abund) and p-value corrected for the structure of the metabolic network (p-struct). (PDF) [file pone.0037919.s003.pdf]

Supplementary Table 2. Summary of microbial functions enriched in diseased or control samples. CLINICAL – indicates whether enrichment occurs in disease or control samples; METHOD – method used to compute significance of enrichment; SIGNIFICANCE – Method-specific assessment of the significance of enrichment: for GSEA [2] we report both the p-value and the q-value obtained by correcting for the False Discovery Rate (FDR); for Metapath [3] we report both the raw p-value for enrichment (p-abund) and a p-value corrected for the structure of the metabolic network (p-struc).

PROCESS	CLINICAL	METHOD	SIGNIFICANCE
**LIPID METABOLISM**			
FATTY ACID DEGRADATION REGULONS	Disease	GSEA	P-val=0.000; FDR Q-val=0.001
LIPID A-ARA4N PATHWAY (POLYMYXIN RESISTANCE)	Disease	GSEA	P-val=0.000; FDR Q-val=0.006
FATTY ACID METABOLISM	Disease	Metapath	P-abund=0.001; P-struc=0.001
FATTY ACID BIOSYNTHESIS	Control	Metapath	P-abund=0.001; P-struc=0.001
GLYCEROPHOPHOLIPID METABOLISM	Disease	Metapath	P-abund=0.001; P-struc=0.02
**AMINO ACID DEGRADATION**			
BRANCHED CHAIN AMINO ACID DEGRADATION REGULONS	Disease	GSEA	P-val=0.000; FDR Q-val=0.000
TRYPTOPHAN CATABOLISM	Disease	GSEA	P-val=0.000; FDR Q-val=0.001
AROMATIC AMINO ACID DEGRADATION	Disease	GSEA	P-val=0.000; FDR Q-val=0.001
CENTRAL META-CLEAVAGE PATHWAY OF AROMATIC COMPOUND DEGRADATION	Disease	GSEA	P-val=0.004; FDR Q-val=0.009
HOMOGENTISATE PATHWAY OF AROMATIC COMPOUND DEGRADATION	Disease	GSEA	P-val=0.000; FDR Q-val=0.001
BUTANOATE AND TYROSINE METABOLISM	Disease	Metapath	P-abund=0.02; P-struc=0.03
HOMOSERINE METABOLISM	Control	Metapath	P-abund=0.001; P-struc=0.001
ASPARTATE AND PURINE METABOLISM	Control	Metapath	P-abund=0.05; P-struc=0.001
**NITROGEN METAOBLISM**			
DENITRIFICATION	Control	GSEA	P-val=0.000; FDR Q-val=0.000
NITRATE AND NITRITE AMMONIFICATION	Control	GSEA	P-val=0.000; FDR Q-val=0.000
OTHER METABOLIC FUNCTIONS			
BENZOATE DEGRIDATION VIA	Disease	Metapath	P-abund=0.01;

HYDROXLATION			P-struc=0.03
DEHYDROGENASE COMPLEXES	Control	GSEA	P-val=0.000; FDR Q-val=0.001
TREHALOSE BIOSYNTHESIS	Control	GSEA	P-val=0.000; FDR Q-val=0.001
GLYCEROL FERMENATION TO 1,3-PROPANEDIOL	Control	GSEA	P-val=0.000; FDR Q-val=0.001
GLUTATHIONE ANALOGS: MYCOTHIOL	Control	GSEA	P-val=0.002; FDR Q-val=0.003
PYRUVATE:FERREDOXIN OXIDOREDUCTASE	Disease	GSEA	P-val=0.000; FDR Q-val=0.000
POLYHYDROXYBUTYRATE METABOLISM	Disease	GSEA	P-val=0.000; FDR Q-val=0.000
ECF CLASS TRANSPORTERS	Disease	GSEA	P-val=0.000; FDR Q-val=0.001
ACETONE BUTANOL ETHANOL SYNTHESIS	Disease	GSEA	P-val=0.000; FDR Q-val=0.001
TERMINAL CYTOCHROME O UBIQUINOL OXIDASE	Disease	GSEA	P-val=0.000; FDR Q-val=0.001
ACETOIN, BUTANEDIOL METABOLISM	Control	GSEA	P-val=0.000; FDR Q-val=0.001
PROTOCATECHUATE BRANCH OF BETA-KETOADIPATE PATHWAY	Disease	GSEA	P-val=0.000; FDR Q-val=0.002
PYRIMIDINE UTILIZATION	Disease	GSEA	P-val=0.000; FDR Q-val=0.002
COBALT-ZINC-CADMIUM RESISTANCE	Disease	GSEA	P-val=0.000; FDR Q-val=0.003
MERCURY RESISTANCE OPERON	Disease	GSEA	P-val=0.000; FDR Q-val=0.006
ACETYL-COA FERMENTATION TO BUTYRATE	Disease	GSEA	P-val=0.000; FDR Q-val=0.006
XYLOSE UTILIZATION	Disease	GSEA	P-val=0.000; FDR Q-val=0.007
CATECHOL BRANCH OF BETA-KETOADIPATE PATHWAY	Disease	GSEA	P-val=0.000; FDR Q-val=0.007
L-ARABINOSE UTILIZATION	Disease	GSEA	P-val=0.003; FDR Q-val=0.007
ALKANESULFONATE ASSIMILATION	Disease	GSEA	P-val=0.000; FDR Q-val=0.007
**NON-METABOLIC FUNCTIONS**			
WHIB AND WHIB-TYPE REGULATORY PROTEINS	Control	GSEA	P-val=0.000; FDR Q-val=0.000
BETA-LACTAMASE CLUSTER IN STREPTOCOCCUS	Control	GSEA	P-val=0.000; FDR Q-val=0.001
FLAVOHAEMOGLOBIN	Control	GSEA	P-val=0.000; FDR Q-val=0.001

DIVERGENT RNA MODIFICATION RELATED CLUSTERS	Control	GSEA	P-val=0.000; FDR Q-val=0.001
A GAMMAPROTEOBACTERIA CLUSTER RELATING TO TRANSLATION	Control	GSEA	P-val=0.000; FDR Q-val=0.001
LATE COMPETENCE	Control	GSEA	P-val=0.000; FDR Q-val=0.001
CONJUGATIVE TRANSPOSON, BACTEROIDALES	Disease	GSEA	P-val=0.000; FDR Q-val=0.000
FLAGELLUM	Disease	GSEA	P-val=0.000; FDR Q-val=0.001
TRANSPOSON RELATED	Disease	GSEA	P-val=0.000; FDR Q-val=0.001
TYPE 4 SECRETION AND CONJUGATIVE TRANSFER	Disease	GSEA	P-val=0.000; FDR Q-val=0.002
