# Supplementary material for: Deep Sequencing of the Oral Microbiome Reveals Signatures of Periodontal Disease
Source: PLoS One. 2012 Jun 4;7(6):e37919. doi: 10.1371/journal.pone.0037919 (PMC3366996; doi:10.1371/journal.pone.0037919)
Supplement: Table S3 — Genomic regions with nucleotide diversity θ values that are more than two standard deviations away from the mean. (PDF) [file pone.0037919.s004.pdf]

Supplementary Table 3. Genomic regions with nucleotide diversity θ values that are more than two standard deviations away from the mean.

Genomic region		Genes
Start	End	
71000	73400	Transcriptional regulator; ABC transporter
191100	192100	GAPDH; Transcriptional regulator
432100	433100	Transcriptional regulator; ABC transporter
574200	575200	Conserved hypothetical protein
640600	642200	Hydrolase; DNA topoisomerase
832200	836300	Fatty acid synthase (Fas)
1531400	1532400	Isocitrate dehydrogenase
1577900	1578900	Conserved hypothetical protein
2093300	2094300	DNA helicase
2277500	2280500	Three ABC transporter proteins
2288500	2292900	Recombination factor protein RarA; Fe-S dehydrogenase; Pullulanase
2538900	2541000	Goadsporin biosynthetic protein
2641700	2642700	Uroporphyrinogen-III synthase
2756700	2759600	ATP-dependent helicase HrpA
