# Supplementary material for: Deep Sequencing of the Oral Microbiome Reveals Signatures of Periodontal Disease
Source: PLoS One. 2012 Jun 4;7(6):e37919. doi: 10.1371/journal.pone.0037919 (PMC3366996; doi:10.1371/journal.pone.0037919)
Supplement: Table S4 — COMBREX [55] functional annotations of new genes predicted from TM7a metagenomic assembly. (PDF) [file pone.0037919.s005.pdf]

Supplementary Table 4. COMBREX [4] functional annotations of new genes predicted from TM7a metagenomic assembly.

Gene ID	Annotation	Best BLAST hit
7_7	Redoxin domain protein(Leptotrichia buccalis C-1013-b)	Lebu_0054(e-value: 9e-87)
16_1	ribonuclease H(Polynucleobacter necessarius subsp. asymbioticus QLW-P1DMWA-1)	rnhA(e-value: 1e-18)
25_1	ATP synthase F1, epsilon subunit(Desulfovibrio desulfuricans subsp. desulfuricans str. ATCC 27774)	Ddes_2189(e-value: 5e-09)
38_0	hypothetical protein(Leptotrichia buccalis C-1013-b)	Lebu_2039(e-value: 7e-52)
61_1	Lipoprotein spr(Cronobacter turicensis)	spr(e-value: 4e-23)
68_1	hypothetical protein(Cronobacter turicensis)	Ctu_37530(e-value: 4e-88)
71_1	transfer protein(Bifidobacterium animalis subsp. lactis AD011)	BLA_0430(e-value: 9e-19)
72_0	putative ATP-dependent Clp protease, proteolytic subunit(Streptococcus suis 05ZYH33)	SSU05_1551(e-value: 7e-16)
73_3	peptide chain release factor-like protein(Bacteroides fragilis NCTC 9343)	prfH(e-value: 6e-06)
88_0	polar amino acid ABC transporter ATP-binding protein(Synechococcus sp. JA-3-3Ab)	CYA_0228(e-value: 1e-33)
111_1	oxidoreductase(Rhodococcus erythropolis PR4)	RER_50690(e-value: 1e-10)
118_1	Na+ transporting oxaloacetate decarboxylase beta chain(Streptococcus pyogenes MGAS10750)	MGAS10750_Spy1053(e-value: 9e-31)
121_0	homoserine dehydrogenase(Thermomicrobium roseum DSM 5159)	trd_0367(e-value: 2e-41)
152_0	SNO glutamine amidotransferase(Leptotrichia buccalis C-1013-b)	Lebu_1507(e-value: 5e-86)
156_0	MATE efflux family protein DinF, truncated(Streptococcus thermophilus CNRZ1066)	str0947(e-value: 1e-29)
207_1	hypothetical protein(Leptotrichia buccalis C-1013-b)	Lebu_0616(e-value: 9e-57)
243_1	uracil-DNA glycosylase(Clostridium difficile R20291)	ung(e-value: 3e-61)
255_0	predicted ORF(Lactobacillus rhamnosus Lc 705)	LC705_01296(e-value: 6e-21)
273_0	binding-protein-dependent transport systems inner membrane component(Geobacillus sp. WCH70)	GWCH70_0296(e-value: 3e-47)
309_0	hypothetical protein(Leptotrichia buccalis C-1013-b)	Lebu_1637(e-value: 7e-102)
313_0	UDP-N-acetylglucosamine 1-carboxyvinyltransferase(Clostridium thermocellum ATCC 27405)	Cthe_0973(e-value: 3e-10)
318_0	cell division protein FtsI/penicillin-binding protein 2(Dictyoglomus thermophilum H-6-12)	DICTH_0339(e-value: 3e-11)
328_1	transcriptional regulator, MarR family(Leptotrichia buccalis C-1013-b)	Lebu_0428(e-value: 8e-71)
355_0	GTP-binding protein TypA/BipA(Streptococcus pyogenes MGAS2096)	MGAS2096_Spy1275(e-value: 7e-17)
356_0	hypothetical protein(Leptotrichia buccalis C-1013-b)	Lebu_0216(e-value: 6e-83)
366_0	hypothetical protein(Pelotomaculum thermopropionicum SI)	PTH_1502(e-value: 5e-07)
372_1	FtsW/RodA/SpoVE family peptidoglycan biosynthesis protein(Desulfotomaculum reducens MI-1)	Dred_0313(e-value: 4e-17)
376_0	glycosyltransferase(Clostridium thermocellum ATCC 27405)	Cthe_0209(e-value: 5e-22)
386_2	phage SPO1 DNA polymerase-related protein(Leptotrichia buccalis C-1013-b)	Lebu_1116(e-value: 1e-76)
421_2	UTP--glucose-1-phosphate uridylyltransferase protein(Nitrosomonas europaea ATCC 19718)	galU1(e-value: 6e-19)

421_5	DNA polymerase III, subunit alpha (dnaE)-like protein(Clostridium perfringens SM101)	CPR_0339(e-value: 2e-91)
441_1	chloride channel protein(Streptococcus equi subsp. zooepidemicus MGCS10565)	Sez_0752(e-value: 4e-07)
467_0	polysaccharide deacetylase family protein(Streptococcus pyogenes MGAS2096)	MGAS2096_Spy1182(e-value: 4e-35)
471_2	tRNA-dihydrouridine synthase(Acinetobacter baumannii ACICU)	ACICU_00749(e-value: 1e-16)
484_1	protein of unknown function DUF74(Leptotrichia buccalis C-1013-b)	Lebu_1999(e-value: 2e-51)
496_0	hypothetical protein(Caldicellulosiruptor saccharolyticus DSM 8903)	Csac_2063(e-value: 4e-08)
497_0	polar amino acid ABC transporter, inner membrane subunit(Leptotrichia buccalis C-1013-b)	Lebu_1162(e-value: 4e-115)
510_1	general secretion pathway protein F(Methylococcus capsulatus str. Bath)	xpsF(e-value: 1e-40)
522_2	GTP-binding protein TypA(Rickettsia bellii RML369-C)	RBE_0285(e-value: 6e-37)
528_0	protein of unknown function DUF1275(Leptotrichia buccalis C-1013-b)	Lebu_0162(e-value: 1e-98)
555_1	hypothetical protein(Cellvibrio japonicus Ueda107)	CJA_2591(e-value: 2e-15)
558_1	putative protease HtpX(Tropheryma whipplei str. Twist)	TWT013(e-value: 3e-82)
574_0	hypothetical protein(Methylacidiphilum infernorum V4)	Minf_1845(e-value: 6e-10)
574_1	Holliday junction DNA helicase RuvB(Vibrio vulnificus CMCP6)	VV1_2161(e-value: 3e-25)
594_0	glycosyl transferase, group 1(Acidithiobacillus ferrooxidans ATCC 53993)	Lferr_0086(e-value: 3e-08)
603_0	glutamine amidotransferase class-I(Leptotrichia buccalis C-1013-b)	Lebu_1909(e-value: 6e-97)
604_0	hypothetical protein(Roseiflexus sp. RS-1)	RoseRS_3685(e-value: 1e-10)
634_0	metalloendopeptidase(Bifidobacterium animalis subsp. lactis AD011)	BLA_1066(e-value: 3e-06)
636_1	transcriptional regulator(Bacillus clausii KSM-K16)	ABC3138(e-value: 1e-25)
646_0	similar to hemolysin-like(Bacillus licheniformis ATCC 14580)	yrkA2(e-value: 2e-15)
666_1	putative cytoplasmic protein(Geobacillus thermodenitrificans NG80-2)	GTNG_3408(e-value: 6e-16)
669_0	phosphotransacetylase(Helicobacter pylori HPAG1)	HPAG1_0885(e-value: 2e-32)
681_0	hypothetical protein(Staphylococcus carnosus subsp. carnosus TM300)	Sca_1875(e-value: 7e-18)
681_1	EmrB/QacA family drug resistance transporter(Bacillus cereus ATCC 10987)	BCE_2916(e-value: 3e-45)
699_1	trans-sulfuration enzyme family protein(Chlorobium tepidum TLS)	CT0703(e-value: 1e-10)
712_0	phosphoglucomutase(Deinococcus radiodurans R1)	pgm(e-value: 2e-07)
712_1	penicillin-binding protein 1A(Staphylococcus aureus RF122)	SAB1588(e-value: 3e-22)
717_1	Glycoside hydrolase family 57(Thermotoga neapolitana DSM 4359)	CTN_1055(e-value: 2e-32)
728_0	PEBP family protein(Geobacter uraniireducens Rf4)	Gura_1648(e-value: 5e-27)
734_2	putative nitrogen regulatory IIA protein (enzyme IIA-ntr) (phosphotransferase enzyme II, A component)(Candidatus Protochlamydia amoebophila UWE25)	ptsN(e-value: 2e-06)
748_9	RNA polymerase sigma-G factor(Clostridium tetani E88)	CTC01128(e-value: 3e-07)
755_1	mannose-1-phosphate guanylyltransferase(Rickettsia bellii RML369-C)	RBE_0245(e-value: 7e-08)
793_0	single-strand binding protein(Leptotrichia buccalis C-1013-b)	Lebu_0211(e-value: 4e-54)
802_0	Tyrosyl-tRNA synthetase (Geobacillus stearothermophilus)	tyrS(e-value: 6e-64)

814_2	elongation factor Ts(Thermus thermophilus HB27)	tsf(e-value: 2e-42)
814_4	30S ribosomal protein S2(Acinetobacter baumannii ATCC 17978)	A1S_2323(e-value: 1e-16)
828_0	narrowly conserved hypothetical protein(Bifidobacterium longum subsp. infantis ATCC 15697)	Blon_1036(e-value: 1e-17)
831_0	ribonuclease P protein component(Leptotrichia buccalis C-1013-b)	Lebu_2305(e-value: 2e-45)
836_4	holliday junction DNA helicase motor protein(Haemophilus parasuis SH0165)	ruvA(e-value: 3e-13)
843_0	Rossmann fold nucleotide-binding protein involved in DNA uptake(Streptococcus suis 05ZYH33)	SSU05_0987(e-value: 8e-16)
851_0	transcriptional regulator(Streptococcus suis 05ZYH33)	SSU05_2193(e-value: 2e-23)
851_1	Signal peptidase I(Prochlorococcus marinus str. MIT 9515)	P9515_05771(e-value: 8e-08)
852_0	truncated resolvase(Staphylococcus aureus subsp. aureus Mu50)	truncated-res(e-value: 2e-13)
855_0	hypothetical protein(Leifsonia xyli subsp. xyli str. CTCB07)	Lxx01200(e-value: 4e-74)
861_1	ribosomal protein L31(Catenulispora acidiphila DSM 44928)	Caci_4370(e-value: 8e-23)
867_0	ammonium transporter(Renibacterium salmoninarum ATCC 33209)	RSal33209_1070(e-value: 2e-08)
903_1	OmpA/MotB domain protein(Leptotrichia buccalis C-1013-b)	Lebu_1720(e-value: 8e-23)
905_0	excinuclease ABC subunit A(Erythrobacter litoralis HTCC2594)	ELI_07895(e-value: 8e-06)
941_0	H+transporting two-sector ATPase E subunit(Leptotrichia buccalis C-1013-b)	Lebu_0755(e-value: 8e-86)
945_0	Spo0B-associated GTP-binding protein(Lysinibacillus sphaericus C3-41)	Bsph_3946(e-value: 5e-50)
954_0	cytidine deaminase(Leptotrichia buccalis C-1013-b)	Lebu_0864(e-value: 4e-64)
964_0	Probable deoxyribose-phosphate aldolase(Thermococcus sibiricus MM 739)	TSIB_1148(e-value: 1e-53)
980_0	hemolysin activator protein precursor(Fusobacterium nucleatum subsp. nucleatum ATCC 25586)	FN0293(e-value: 8e-10)
1072_0	Putative AAA+ superfamily ATPase(Bifidobacterium longum DJO10A)	BLD_0142(e-value: 9e-19)
1090_1	hypothetical protein(Leptotrichia buccalis C-1013-b)	Lebu_1795(e-value: 1e-44)
1092_2	uroporphyrinogen III synthase (hemD)(Methanocaldococcus jannaschii DSM 2661)	MJ0994(e-value: 1e-13)
1092_3	uroporphyrinogen-III C-methyltransferase(Lysinibacillus sphaericus C3-41)	Bsph_3433(e-value: 2e-15)
1095_0	hypothetical protein(Streptococcus equi subsp. zooepidemicus MGCS10565)	Sez_1955(e-value: 9e-09)
1138_2	hypothetical protein(Leptotrichia buccalis C-1013-b)	Lebu_1783(e-value: 4e-45)
1141_0	branched-chain amino acid transport protein(Bacillus cereus E33L)	azlC(e-value: 1e-13)
1187_4	hypothetical protein(Leptotrichia buccalis C-1013-b)	Lebu_1940(e-value: 7e-65)
1285_1	PTS system, mannose-specific IIAB component(Streptococcus pyogenes MGAS2096)	MGAS2096_Spy1506(e-value: 2e-33)
1306_0	riboflavin kinase(Bacillus cereus 03BB102)	BCA_2148(e-value: 5e-17)
1319_0	NUDIX hydrolase(Pyrobaculum arsenaticum DSM 13514)	Pars_2356(e-value: 5e-08)
1323_8	heat shock protein Hsp20(Sulfurihydrogenibium sp. YO3AOP1)	SYO3AOP1_1159(e-value: 5e-13)
1323_9	phosphoribosyltransferase(Candidatus Solibacter usitatus Ellin6076)	Acid_3419(e-value: 1e-12)
1365_3	ribosomal protein L33(Leptotrichia buccalis C-1013-b)	Lebu_1899(e-value: 2e-21)
1426_1	Dihydrolipoyllysine-residue succinyltransferase(Leptotrichia buccalis C-1013-b)	Lebu_0543(e-value: 3e-140)

1468_2	putative metal dependent phosphohydrolase(Leptotrichia buccalis C-1013-b)	Lebu_1403(e-value: 1e-49)
1473_5	hypothetical protein(Listeria monocytogenes EGD-e)	lmo2078(e-value: 7e-21)
1531_0	50S ribosomal protein l14(Heliobacterium modesticaldum Ice1)	rplN(e-value: 2e-43)
1561_4	TfoX domain protein(Leptotrichia buccalis C-1013-b)	Lebu_1291(e-value: 3e-52)
1604_0	phosphoribosyl-ATP pyrophosphatase(Lactobacillus fermentum IFO 3956)	hisE(e-value: 3e-25)
1616_0	30S ribosomal protein S8(Pelobacter propionicus DSM 2379)	rpsH(e-value: 3e-26)
1619_1	hypothetical protein(Staphylococcus saprophyticus subsp. saprophyticus ATCC 15305)	SSP0469(e-value: 1e-07)
1634_0	ABC type periplasmic choline/glycine/betaine binding protein(Yersinia pestis Nepal516)	YPN_2780(e-value: 8e-08)
1640_1	ribosomal protein L15(Coprothermobacter proteolyticus DSM 5265)	rplO(e-value: 1e-19)
1646_0	hypothetical protein(Leptotrichia buccalis C-1013-b)	Lebu_1518(e-value: 5e-113)
1674_0	MarR family transcriptional regulator(Streptococcus agalactiae A909)	SAK_1868(e-value: 9e-39)
1685_0	hypothetical protein(Macrococcus caseolyticus JCSC5402)	MCCL_1596(e-value: 9e-24)
1695_0	hypothetical protein(Yersinia pestis Nepal516)	YPN_2781(e-value: 5e-11)
1707_0	sodium-glutamate/aspartate symporter(Bacillus halodurans C-125)	BH3227(e-value: 5e-10)
1710_1	membrane protein of unknown function(Delftia acidovorans SPH-1)	Daci_0859(e-value: 2e-08)
1713_1	50S ribosomal protein L5(Psychrobacter sp. PRwf-1)	rplE(e-value: 6e-44)
1717_2	mannose-1-phosphate guanylyltransferase, putative(Clostridium novyi NT)	NT01CX_0602(e-value: 3e-12)
1718_0	NUDIX hydrolase(Novosphingobium aromaticivorans DSM 12444)	Saro_2655(e-value: 3e-06)
1728_0	hypothetical protein(Bacillus cereus ATCC 10987)	BCE_4466(e-value: 1e-13)
1729_0	hypothetical protein(Actinobacillus pleuropneumoniae serovar 7 str. AP76)	APP7_0688(e-value: 1e-15)
1735_0	50S ribosomal protein L6(Bradyrhizobium sp. BTAi1)	rplF(e-value: 8e-40)
1736_0	phenazine biosynthesis protein PhzF family(Akkermansia muciniphila ATCC BAA-835)	Amuc_0023(e-value: 8e-81)
1740_0	hypothetical protein(Methanosphaera stadtmanae DSM 3091)	Msp_0734(e-value: 2e-20)
1754_1	Protein of unknown function DUF214(Brucella abortus S19)	BAbS19_I15750(e-value: 3e-22)
1754_3	putative ABC transporter, ATP-binding protein(Streptococcus mutans UA159)	SMU.863(e-value: 8e-68)
1760_0	ribosome-binding factor A(Leptotrichia buccalis C-1013-b)	Lebu_1316(e-value: 3e-58)
1766_1	hypothetical protein(Leptotrichia buccalis C-1013-b)	Lebu_1144(e-value: 1e-39)
1769_1	30S ribosomal protein S10(Lactobacillus reuteri JCM 1112)	LAR_1395(e-value: 1e-31)
1773_0	lipid A biosynthesis acyltransferase(Leptotrichia buccalis C-1013-b)	Lebu_0285(e-value: 1e-143)
1776_2	UDP-N-acetylmuramate--L-alanine ligase(Burkholderia cenocepacia MC0-3)	murC(e-value: 5e-44)
1777_1	malate dehydrogenase(Lactobacillus rhamnosus GG)	LGG_02876(e-value: 2e-27)
1778_1	hypothetical protein(Leptotrichia buccalis C-1013-b)	Lebu_0532(e-value: 1e-36)
1780_0	D-tyrosyl-tRNA(Tyr) deacylase(Leptotrichia buccalis C-1013-b)	Lebu_0701(e-value: 2e-55)
1783_1	hypothetical protein(Chlamydophila pneumoniae J138)	CPj0340(e-value: 2e-09)
1784_1	hypothetical protein(Porphyromonas gingivalis ATCC 33277)	PGN_0382(e-value: 5e-21)
1788_1	small GTP-binding protein(Leptotrichia buccalis C-1013-b)	Lebu_1928(e-value: 1e-85)

1798_0	Type IV pili nucleotide-binding protein(Francisella tularensis subsp. holarctica)	FTL_1770(e-value: 2e-53)
1800_1	ParA family ATPase(Symbiobacterium thermophilum IAM 14863)	STH3333(e-value: 2e-76)
1801_0	single-stranded-DNA-specific exonuclease(Streptococcus pyogenes MGAS9429)	MGAS9429_Spy0784(e-value: 5e-43)
1804_1	recombinase(Fusobacterium nucleatum subsp. polymorphum ATCC 10953)	FNP_pFN3g09(e-value: 2e-26)
1805_1	hypothetical protein(Flavobacterium psychrophilum JIP02/86)	FP0837(e-value: 3e-35)
1811_1	ribosomal protein S9(Petrotoga mobilis SJ95)	Pmob_0800(e-value: 2e-31)
1812_0	ribonucleotide-diphosphate reductase subunit beta(Candidatus Liberibacter asiaticus str. psy62)	CLIBASIA_00075(e-value: 4e-16)
1813_0	phosphotransferase system IIC component, glucose/maltose/N-acetylglucosamine-specific(Streptococcus suis 98HAH33)	SSU98_1936(e-value: 2e-68)
1814_0	dihydrolipoamide dehydrogenase(Desulfotomaculum reducens MI-1)	Dred_2832(e-value: 5e-36)
1821_11	hypothetical protein(Clostridium kluyveri NBRC 12016)	CKR_1146(e-value: 5e-09)
1828_4	phage protein(Flavobacterium psychrophilum JIP02/86)	FP2178(e-value: 1e-12)
1829_0	5-methyltetrahydropteroyltriglutamate--homocysteine methyltransferase(Lysinibacillus sphaericus C3-41)	Bsph_2979(e-value: 9e-65)
1833_0	rRNA methylase(Micrococcus luteus NCTC 2665)	Mlut_02030(e-value: 1e-54)
1842_0	ABC-type polysaccharide/polyol phosphate transport system, ATPase component(Streptococcus suis 98HAH33)	SSU98_1301(e-value: 9e-86)
1842_1	ABC-type polysaccharide/polyol phosphate export system, permease component(Streptococcus suis 98HAH33)	SSU98_1302(e-value: 9e-66)
1843_0	FolD bifunctional protein(Thermodesulfovibrio yellowstonii DSM 11347)	THEYE_A0674(e-value: 1e-33)
1844_0	CTP synthase(Mycoplasma mycoides subsp. mycoides SC str. PG1)	pyrG(e-value: 3e-40)
1844_1	large conductance mechanosensitive channel protein(Dyadobacter fermentans DSM 18053)	Dfer_3491(e-value: 4e-06)
1845_1	chaperonin Hsp40(uncultured methanogenic archaeon RC-I)	dnaJ(e-value: 7e-51)
1850_1	hypothetical protein(Thermobifida fusca YX)	Tfu_0647(e-value: 4e-24)
1850_2	hypothetical protein(Corynebacterium aurimucosum ATCC 700975)	cauri_2344(e-value: 1e-24)
1853_1	ribosomal protein S4(Chloroflexus sp. Y-400-fl)	Chy400_2582(e-value: 3e-48)
1853_4	ribosomal protein L17(Nautilia profundicola AmH)	rplQ(e-value: 4e-28)
1889_2	ybaK/ebsC protein(Leptotrichia buccalis C-1013-b)	Lebu_0669(e-value: 8e-71)
1968_2	ABC transporter, permease; probable glutamine ABC transporter, permease(Bacillus cereus Q1)	glnP(e-value: 1e-17)
2054_1	glycosyl transferase, group 1(Acaryochloris marina MBIC11017)	AM1_2714(e-value: 7e-13)
2133_1	protein of unknown function DUF45(Prosthecochloris aestuarii DSM 271)	Paes_0312(e-value: 1e-09)
